# Pharmaceutical Equivalence of Film-Coated and Chewable Tablets: A Comparative Dissolution Study Using Pulverized Chewable Tablets

**DOI:** 10.3390/ph17111525

**Published:** 2024-11-12

**Authors:** Suck-Yong Park, Sung-Up Choi

**Affiliations:** 1 Department of Pharmaceutical Engineering, Cheongju University, Cheongju 28503, Republic of Korea; 2 Central Research Center, Introbiopharma, Pyeongtaek-si 17702, Republic of Korea

**Keywords:** pharmaceutical equivalence, reference for chewable tablet, pulverized chewable tablet, dissolution profile, famotidine

## Abstract

Famotidine is a histamine H2 receptor antagonist used in the treatment of gastrointestinal disorders. It is available in multiple formulations, including film-coated tablets, chewable tablets, oral suspension, and injections. The purpose of this study was to develop and evaluate the film-coated tablet (FT) containing famotidine, magnesium hydroxide, and precipitated calcium carbonate, designed to be pharmaceutically equivalent to the marketed chewable tablet (CT). To achieve the pharmaceutical equivalence of two tablets, the dissolution profiles of FT should be similar to those of CT. However, since CT is intended to be chewed before swallowing, testing it in its intact form would not provide accurate results. Therefore, pulverized chewable tablets (PCT) were used as the reference product. The dissolution, performed by the paddle method at 50 rpm, was analyzed by the validated UV method. Similarity factor (*f*_2_) and difference factor (*f*_1_) were calculated to assess the equivalence of the dissolution profiles. The results demonstrated that the dissolution profiles of the FT and CT were similar. Additionally, the acid-neutralizing capacity test confirmed the equivalence of the two antacids. This study is one of the first to propose that dissolution tests for pharmaceutical equivalence should be conducted on pulverized CTs when developing generic equivalents to CTs.

## 1. Introduction

Gastric disease is one of the most common diseases among Koreans. The mortality rate of gastric cancer has shown a decrease of nearly 5% on average over the past 10 years according to the National Cancer Information Center’s analysis of cancer incidence trends, yet there are still 13.9 patients per 100,000 people dying annually on average [1], making it the fourth most frequent occurrence and accounting for 10% of all cancer types. Especially among males, it is the second most frequently occurring disease, at a rate of 76.3 per 100,000 people according to the Korean Statistical Information Service’s cancer incidence rate for 24 types of cancer in December 2023, with gastric cancer mortality rates being the highest among individuals in their 30s. The most common diseases among gastric diseases are gastritis, duodenitis, and gastroesophageal reflux disease. Gastritis is a very common disease in Korea. Pharmacological treatments for gastritis include acid suppressants, mucosal protectants, H2 receptor antagonists, proton pump inhibitors, potassium-competitive acid blockers (P-CABs), antispasmodics, and analgesics. However, acid suppressants and H2 receptor antagonists remain the primary therapeutic options due to their cost-effectiveness and proven efficacy.

In Korea, over the past 20 years, H2 receptor antagonists, such as ranitidine, and mucosal protectants have experienced significant growth in the domestic gastrointestinal disease market. However, sales of ranitidine were discontinued due to its association with NDMA(N-Nitrosodimethylamine) carcinogenic incidents. Although the need for alternative drugs has since been recognized, novel research on ranitidine alone or its combination drug has struggled due to the cost of regulatory approval and the emergence of newer drugs such as P-CABs.

The domestic market for H2 receptor antagonists includes famotidine, nizatidine, lafutidine, cimetidine, and roxatidine. Since the withdrawal of ranitidine, famotidine and nizatidine have taken its place. The standard dose of famotidine is 10–20 mg, relatively lower than other H2 receptor antagonists, making it suitable for combination formulations. In Korea, the combination formulation of H2 receptor antagonists and acid suppressants was first developed with cimetidine 50 mg. In 1998, a combination formulation of cimetidine/magnesium aluminosilicate/hydrotalcite/sodium bicarbonate was approved. Later, the combination formulation of famotidine and acid suppressants was first approved as Famocomp^®^ tablets by GL Pharmaceuticals in 2003. This composition consists of famotidine/precipitated calcium carbonate/magnesium hydroxide, which is identical to the Pepcid^®^ Complete chewable tablet marketed in the United States. Using 10 mg of famotidine allows for a relatively small tablet size. However, chewable tablets are less preferred by adults in Korea, resulting in a smaller market size. Only eight active pharmaceutical ingredients are available as a single-ingredient drug in Korea (Table 1). Most of them are used for asthma, antihistamines, and erectile dysfunction. The majority of combination products consist of vitamins, with some antihistamines and gastrointestinal medications.

On the other hand, in vitro dissolution comparisons of regular tablets with chewable tablets have been performed either as intact chewable tablets [2] or crushed [3,4]. Since chewable tablets are designed to be swallowed after chewing, comparative dissolution with intact chewable tablets is not appropriate, considering the administration method of chewable tablets. Dissolution tests compared with crushed chewable tablets also did not specify the size of the crushed particles.

This study aimed to develop over-the-counter generic tablets (film-coated tablet, FT), not chewable tablets, which are undesirable to Korean adults, combining famotidine as an H2 receptor antagonist, and precipitated calcium carbonate and magnesium hydroxide as acid suppressants, allowing patients to self-treat at a relatively low cost. To evaluate the pharmaceutical equivalence, we pulverized the chewable tablet (CT), sieved with 12-mesh screens, and used it as the reference drug for the comparative dissolution test.

## 2. Results

### 2.1. Preparation and Characterization of Tablets

Film-coated tablets (FT) were manufactured, with dimensions of 19.8 mm in length and 8.7 mm in width. The assay for famotidine content was analyzed by the validated HPLC method, which is described in the official monograph (Korean Pharmacopoeia 12, KP). The assay for FT and CT (marketed chewable tablets, the reference drug) was 97.1 ± 0.51% and 96.4 ± 0.38%, respectively. The disintegration time was 4 min and 55 s for FT and 2 min and 10 s for CT. The hardness for FT and CT was 316.7 ± 9.2 N and 133.5 ± 7.9 N, respectively.

### 2.2. Method Validation for Dissolution Test

**Figure 1:**
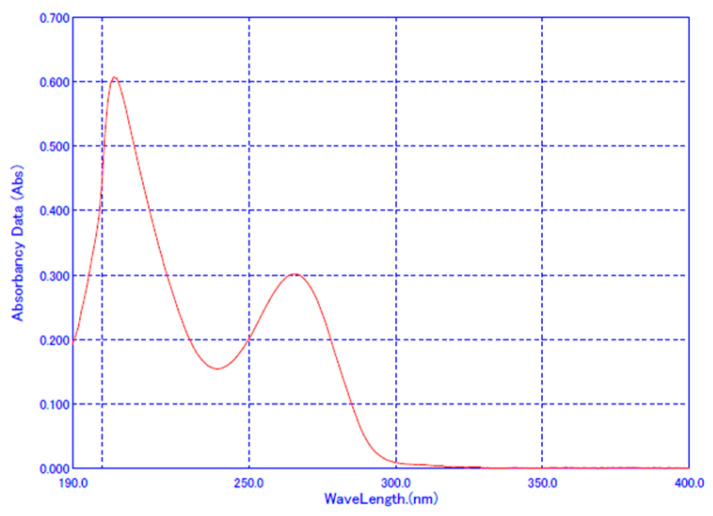
UV spectrum of famotidine.

The UV spectrum of famotidine is shown in Figure 1, and the wavelength of maximum absorbance was revealed to be 265 nm, which conforms to KP. The system suitability test satisfied the criteria with a relative standard deviation (RSD) of 0.07% to 0.18% for the analysis of the standard solution at the same concentration in each dissolution test solution (Table 2). Specificity was confirmed by the absorbance of the placebo and the percentage recovery. No interference from the medium and placebo was observed.

Linearity was achieved in each dissolution medium (Table 3). The coefficient of determination (R^2^) ranged from 0.9978 to 1.0000. Linear relationship (y = 0.0310 x + 0.0010, R^2^ = 1.0000) was obtained with a concentration range of 0.509 to 12.212 µg/mL. Accuracy was evaluated at the lowest, intermediate, and highest concentrations, with the percent recovery ranging from 98.39% to 99.43%. Repeatability (intra-assay precision) and laboratory precision (reproducibility) showed a RSD of 0.47~2.00% and 0.51~1.64%, respectively. The detection limits (DLs) and quantification limits (QLs) in four different media were 0.042 to 0.157 µg/mL and 0.130 to 0.478 µg/mL, respectively. Therefore, the established UV analysis method was well validated and used to analyze the dissolution comparison.

### 2.3. Dissolution Profiles

The content of the reference drug and the test drug analyzed by the HPLC method was 96.4% and 97.1%, respectively, meeting the content criteria (90.0% to 110.0%). Since the content difference was within 5%, it conforms to the Standard on Pharmaceutical Equivalence Test criteria by the Korea Ministry of Food and Drug Safety (KMFDS) [5]. In an in vitro comparative test for acid-neutralizing capacity (KP) for two other active ingredients, such as precipitated calcium carbonate and magnesium hydroxide, both the reference drug and the test drug met the criteria by yielding 210 mL and 218 mL, respectively, which is greater than or equal to 200 mL.

**Figure 2:**
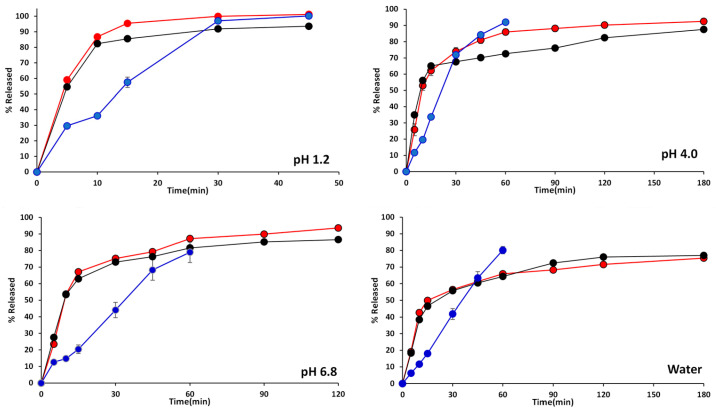
Comparative release profiles of the test drugs (red circle), the pulverized reference drugs (black circle), and the intact chewable reference drugs (blue circle).

Figure 2 demonstrates the dissolution profiles of the test drug (FT), the reference drug (CT), and the pulverized reference drug (PCT). In all test media, the dissolution profiles of FT and PCT revealed similar patterns, while those of CT showed relatively low and delayed dissolution results, especially up to 30 min of the early stage of dissolution. In pH 1.2, both FT and PCT demonstrated over 80% dissolution within 10 min. Disintegration was the rate-limiting step that determined dissolution. The intact chewable tablet (CT) was found to release only 36% at 10 min in pH 1.2 while FT and PCT released over 80%. Since CT is in chewable tablet form and needs disintegration time, its initial amount released was relatively low. However, after disintegration, its dissolution profile did not show a significant difference compared to FT.

### 2.4. Difference Factor (f_1_) and Similarity Factor (f_2_)

Using the average dissolution rates of the test (FT) and reference drugs (PCT, pulverized reference drug), the difference factor (*f*_1_) and similarity factor (*f*_2_) were calculated (Table 4). For each test solution, the f1 values for FT and PCT ranged from 4.28 to 10.03, while the *f*_2_ values ranged from 53.10 to 75.95. Since the *f*_2_ values equal to or greater than 50 are recognized as similar, the two dissolution curves from FT and PCT were revealed as pharmaceutical equivalence.

## 3. Discussion

### 3.1. Selecting an Appropriate Form as the Reference Drug for the Pharmaceutical Equivalence

In order to obtain approval from the regulatory authority for all generic drugs on the market, pharmaceutical equivalence must be achieved according to the Standards of the KMFDS. The chewable tablets are designed to be chewed before swallowing, and the chewing process involves breaking the tablet into smaller pieces. Once chewed, the active ingredients are absorbed into the body through a process similar to that of regular tablets. Therefore, a dissolution test using a chewable tablet in its original form may produce significantly different results compared to the dissolution profiles of its small fragments post-chewing, potentially providing inaccurate information about the actual efficacy of the formulation. As shown in the dissolution graphs in Figure 2, the dissolution patterns of the reference chewable drug (CT) and its pulverized reference drug (PCT) differ significantly. The dissolution rate of PCT was significantly faster than CT, as the pulverized tablet increases its surface area, enhancing contact with the dissolution medium and accelerating the dissolution process.

Therefore, for a pharmaceutical company aiming to develop a tablet formulation equivalent to the marketed chewable tablets as a reference, selecting the appropriate form—whether intact, crushed, or pulverized—of the chewable tablets for comparative dissolution tests could be a critical challenge. Since no one swallows the chewable tablet, the dissolution profiles of the test drug should be compared to the pulverized form of the reference drug after chewing the tablet. After swallowing a pulverized form, the small particles are exposed to the gastrointestinal environment, where further disintegration and dissolution occur. Therefore, it is more appropriate to compare the dissolution of the pulverized particles, rather than intact chewable tablets, with that of regular tablets (see Table 5).

In bioequivalence studies with chewable tablets (Table 5), pharmaceutical equivalence was evaluated by taking the intact chewable tablets with water [2,10,15] by chewing them and comparing them to tablets [13,14]. Even in the study of taking chewed tablets, there was no further description of particle size.

In the case of dissolution tests, as with bioequivalence studies, some studies utilized intact chewable tablets [2], and some used pulverized tablets after crushing the chewable tablets [3,4]. Nevertheless, the results of dissolution may vary considerably depending on whether the chewable tablet is used as an intact tablet or chewed. While the research papers used terms such as ‘crushed’, ‘fine powder’, ‘ground’, and ‘pulverized’, there was no further explanation of what particle range they referred to. Particle size seems to refer to smaller sizes in the following order, but nothing is scientifically defined: intact > crushed > pulverized. It is also unclear what particle size is specifically meant by the terms ‘fine’ or ‘ground’. In any case, a regulatory guideline appears to be warranted. FDA guidance for chewable tablets mentioned the distinction between intact and chewed tablets but did not elaborate on the specific method for comparative dissolution test [18].

Hence, we searched for published papers to find out the proper size of the pulverized particle for the comparative dissolution test. According to the literature on the reliability of masticatory efficiency for peanuts [19], after thorough chewing, the passage rates through sieves of sizes 2.0 mm, 1.0 mm, 0.5 mm, and 0.25 mm were approximately 76.7%, 55.2%, 42.6%, and 38.0%, respectively. It was reported that the majority of particles passed through the 2.0 mm sieve, with 55.2% passing through the 1.0 mm sieve. Another study [20] revealed the particle sizes of peanuts after 25 chewings, with sizes above 2.0 mm accounting for 14.6% and below 2.0 mm accounting for 85.4%. Particularly, particles smaller than 0.5 mm accounted for 61.5% of the total, indicating that the most of particles were smaller than 0.5 mm.

**Figure 3:**
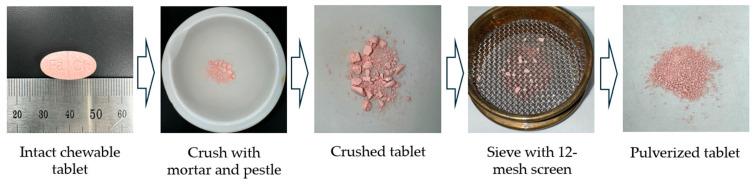
Preparation of pulverized tablet.

In the pharmaceutical field, mesh has been used as the standard sieve unit. According to KP, a 12-mesh sieve has a pore size of 1.4 mm. Therefore, the comparative dissolution test was performed in this study using a pulverized reference chewable tablet passed through a 12-mesh sieve (Figure 3).

Upon comparison of the dissolution patterns between the pulverized reference drug and the intact chewable reference tablet, it was observed that the dissolution rate of the intact reference tablet was significantly lower during the initial 30 min.

As shown in Figure 2, since the amount released from the pulverized tablet and the intact chewable tablet were significantly different, the selection of the physical form of the target drug for generics would be critical. As described above, we believe that the pulverized form of the chewable tablet should be used as the reference drug for generic drugs. When developing tablets with the same active pharmaceutical ingredients as chewable tablets, no guidelines have been found so far on whether the intact form of the chewable tablet or the pulverized form should be used as a reference drug.

### 3.2. Determining Dissolution Similarity

Famotidine is a weak base with a pKa of approximately 6.76 at 23 °C [21]. In acidic solutions such as glacial acetic acid and pH 1.2 medium, famotidine exists in the protonated form; therefore, its solubility is increased. Although famotidine is classified as a poorly soluble drug, the solubility in acetic acid is 500 mg/mL, while in water it iss 1 mg/mL [21,22]. In the dissolution tests, the amount of famotidine released at pH 1.2 exceeds 90% within 30 min, whereas it does not reach 60% in water within the same time frame (Figure 2). These findings align with the established relationship between pKa and solubility, where the ionization state of famotidine significantly influences its solubility across different pH ranges. Since an aqueous solubility of famotidine was as low as 2.7 mM at 23 °C [22], the amount of famotidine released in water showed the lowest results among the four media tested. The time for reaching 80% of release from the pulverized reference drug (PCT) was 10 min at pH 1.2, 45 min at pH 4.0, 60 min at pH 6.8, and over 180 min in water (Figure 2). Based on the pKa of famotidine, the ionization profile suggests that the dissolution rate at pH 4.0 should exceed that observed at pH 6.8. However, experimental data demonstrate comparable dissolution rates under both conditions. This discrepancy can be explained by several contributing factors, including the polar structure of famotidine, weak ionic interactions, dynamic pH shifts during the process of solubilization, salting-out effects, and interactions among ions in the buffer system [21]. Nevertheless, two dissolution graphs of FT and PCT in all media revealed similar profiles.

Currently, the criteria for determining pharmaceutical equivalence in Korea include biological equivalence tests, comparative dissolution tests, comparative disintegration tests, and physicochemical equivalence tests [5]. Biological equivalence tests are mandatory for the development of all generic pharmaceuticals. However, in certain cases, bioequivalence studies may be exempted for non-prescription combination drugs. Combination drugs containing famotidine, magnesium hydroxide, and precipitated calcium carbonate are already approved and marketed in the United States and Korea. Therefore, when developing non-prescription drugs with the same composition, it is possible to be exempt from the costly biological equivalence tests. Instead, comparative dissolution tests must be conducted to ensure pharmaceutical equivalence. The efficacy of the antacid components, such as magnesium hydroxide, and precipitated calcium carbonate can be confirmed through comparative disintegration and antacid tests, while only famotidine needs to be compared through comparative dissolution tests. Equivalence in comparative dissolution tests is determined based on the similarity factor (*f*_2_). If the similarity factor is 50 or greater, the dissolution profiles of the two formulations are considered comparable, indicating that the formulations can be regarded as equivalent [5,23].

On the other hand, the difference factor (*f*_1_) represents the relative error between two dissolution profiles, and if it is between 0 and 15, the two dissolution profiles are considered to be similar [24].

In this study, the dissolution similarity was compared between the test drugs (FT) and pulverized reference drugs (PCT), as well as between PCT and the intact reference chewable tablets (CT). The difference factors for FT and PCT in all four test conditions were below 10.03 (Table 4), indicating no significant difference in dissolution profiles between the two drugs. The similarity factors ranged from 53.10 to 75.95, indicating that the two graphs are similar. Therefore, it can be concluded that the dissolution profiles of FT and PCT are similar based on the difference and similarity factor assessments.

However, the difference factors for PCT and CT exceeded the threshold value of 15, and the similarity factors were below 50, failing to meet the criteria. Since CT requires disintegration time, the initial dissolution was delayed compared to PCT, resulting in a different dissolution profile.

The FDA has issued guidance on bioequivalence testing for chewable tablets that require the tablet to be chewed and then swallowed without water [25]. Currently, there are no specific regulations for comparative dissolution between chewable tablets and regular tablets. However, if authorities review these cases, they may need to consider whether to use an intact chewable tablet or a pulverized tablet as the reference drug. In addition, since variations in particle size of the pulverized tablets can lead to differences in dissolution profiles, the establishment of appropriate guidelines to address this issue should be discussed.

## 4. Materials and Methods

### 4.1. Materials

Famotidine (100.3%), magnesium hydroxide (98.0%), and precipitated calcium carbonate (99.7%) were purchased from Rakshit Pharmaceuticals Ltd. (Andhra Pradesh, India), Il-Yang Pharmaceutical Co., Ltd. (Yongin, Republic of Korea), and Shanghai Nuocheng Pharmaceutical Co., Ltd. (Shanghai, China), respectively. Dextrose monohydrate (Pharmatose^®^ 200M, DFE Pharma), corn starch (Roquette, Lestrem, France), low-substituted hydroxypropylcellulose (L-HPC, LH11, Sin-Etsu, Tokyo, Japan), magnesium stearate (Faci, Jurong Island, Singapore), colloidal silicon dioxide (Aerosil^®^ 200, Degussa, Rheinfelden, Germany), and Opadry^®^ Green (03B41083, Colorcon, PA, USA) were purchased. The reference drug was Famocomp^®^ Chewable Tablet (Lot. FRT20001, GL Pharmaceuticals, Anyang, Republic of Korea). The additives used in the reference drug are as follows: D-mannitol, lactose monohydrate, microcrystalline cellulose, hydroxypropyl cellulose, sucralose, colloidal silicon dioxide, magnesium stearate, and peppermint flavor. All other reagents for analysis were used as received without further treatment.

### 4.2. Preparation of Famotidine Fixed-Dose Tablets (FT) with Antacids

Film-coated tablets (FT, Lot. K0901, Introbiopharma, Pyeongtaek, Republic of Korea) containing famotidine 10 mg, magnesium hydroxide 165 mg, and precipitated calcium carbonate 800 mg were manufactured by a wet granulation method. Briefly, magnesium hydroxide, precipitated calcium carbonate, and lactose were mixed and then granulated by adding a starch- and water-binder solution. After drying with a fluid-bed dryer, famotidine, lactose, and L-HPC were added and mixed. Finally, colloidal silicon dioxide and magnesium stearate were added for the final mixing, and this mixture was used for tableting. The mass of each tablet was adjusted to 1240 mg. The tablets were processed using a rotary tablet press machine (ZP-S, STC, Liaocheng DR Machinery, Liaocheng, China), and the tablet-coating process was conducted using an SFC-30F (Sejong Pharmatech, Incheon, Republic of Korea).

### 4.3. Quality Control of the Tablets

The hardness of the tablets was measured using an Erweka TBH 125 (Langen, Germany) on 10 tablets, while the disintegration test was conducted using a KDIT-200 (Kukje Engineering, Paju, Republic of Korea). Dissolution was carried out using a DS-14000 (Labindia, Thane, India), with 12 tablets of the test (FT) and reference drug (CT) each, according to the method in the Korean Pharmacopoeia 12th ed. (KP), the paddle method, at 37 ± 0.5 °C, 50 rpm, and 900 mL of test solutions. Test solutions included water, pH 1.2 (hydrochloric acid and sodium chloride), pH 4.0 (acetate), and pH 6.8 (phosphate) buffer solutions for dissolution comparison.

Content uniformity was performed using HPLC (Waters e2695, Waters, MA, USA). The analytical conditions referenced KP, with a detection wavelength of 254 nm, a C18 column (4.6 × 150 mm, 5 µm), and a mobile phase consisting of acetonitrile:methanol:water (1-heptanesulfonic acid sodium salt 2 g dissolved in 900 mL water, adjusted to pH 3.0 with acetic acid (100), then diluted to 1000 mL with water) (240:40:1000). The flow rate was adjusted to achieve a retention time of approximately 6 min for famotidine.

The analysis of famotidine for dissolution was performed at the maximum absorption wavelength of famotidine, 265 nm, using a UV Spectrophotometer (V-730, Jasco, Tokyo, Japan) according to KP. Method validation was performed for each test solution.

The acid-neutralizing capacity of the acid suppressants (magnesium hydroxide, precipitated calcium carbonate) was evaluated using the General Test Method specified in the KP [26]. The acid-neutralizing capacity of the acid suppressants (magnesium hydroxide, precipitated calcium carbonate) was evaluated using the General Test Method specified in the KP [26]. The acid-neutralizing capacity evaluates a pharmaceutical preparation’s capacity to neutralize gastric acid. Briefly, accurately weigh and finely powder at least 10 tablets (test or reference). Transfer a portion of the powder, equivalent to approximately 200 mg of precipitated calcium carbonate, into a 200 mL flask. Add 100 mL of 0.1 mol/L HCl, seal, and shake for 1 h at 37 ± 2 °C. Filter and cool the solution, then take 50 mL of the filtrate and titrate the remaining HCl with 0.1 mol/L NaOH volumetric solution (VS) to a pH of 3.5. Repeat the same procedure with a blank sample.

Acid-neutralizing capacity (mL) = (*b* − *a*) *f* × 2 × *t*/*s* (1)

*a*: Consumed amount of 0.1 mol/L sodium hydroxide VS (mL).
*b*: Consumed amount of 0.1 mol/L sodium hydroxide VS in blank test (mL).
*f*: Normality factor of 0.1 mol/L sodium hydroxide VS.
*t*: Weight of a tablet (mg).
*s*: Sample quantity (mg).

### 4.4. Method Validation for the UV Analysis of Dissolution

For the dissolution testing, UV analysis method validation was conducted for each test solution, evaluating parameters such as specificity, linearity, range, accuracy, precision, detection limit, and quantification limit. Standard stock solutions were prepared at a concentration of 200 µg/mL using each test solution, and system suitability was determined by repeating the analysis of the same concentration six times and calculating the relative standard deviation (RSD).

Linearity was confirmed by preparing calibration curves in the range of 0.5 to 12 µg/mL, corresponding to 5 to 110% of famotidine upon full release of the drug. Specificity was conducted using the test solutions, where placebo samples contained all components except the active ingredient. Accuracy and precision were assessed based on percentage recovery and relative standard deviation (RSD) at low, intermediate, and high concentrations.

Detection (DL) and quantification limit (QL) were calculated from the standard deviation of the response (σ) and the slope of the calibration curve (*S*) using the following equations derived from the linearity test data.

DL = 3.3 × σ/*S* (2)

QL = 10 × σ/*S* (3)

### 4.5. Dissolution Comparison with Difference Factor (f_1_) and Similarity Factor (f_2_)

The comparative dissolution test was performed according to the Standard on Pharmaceutical Equivalence Test (KMFDS 2021). Twelve tablets were tested by the paddle method (50 rpm, 37 ± 0.5 °C) in four different dissolution media (pH 1.2, 4.0, 6.8, and water). The test drug was used in intact tablet form (FT), while the reference drug was used in pulverized form (PCT) sieved with 12-mesh screens after crushing the tablets (Figure 3). The intact reference drug (CT) was also tested.

The similarity between the dissolution profiles of the test and reference drugs was assessed by calculating the difference factor (*f*_1_) and similarity factor (*f*_2_) based on their dissolution graphs. The similarity was determined by calculating *f*_2_ using the following equation, according to the guidelines of the Ministry of Food and Drug Safety of Korea (KFMDS) and the formula proposed by Shah et al. in 1998 [24]:

(4) $$f_{2}=50\times log\;\{[1+\frac{1}{n}\;\sum_{t=1}^{n}{{\left(R_{t}-T_{t}\right)}^{2}]}^{-0.5}\times 100\}\;$$

where:

*R_t_* = the dissolution value of the reference drug at time *t.*
*T_t_* = the dissolution value of the test drug at time *t.*
*n* = the number of time points.
*t* = time point.

The *f*_2_ value ranges between 0 and 100, and a higher value indicates greater similarity between the dissolution profiles of the test and reference drugs.

The difference factor (*f*_1_) was calculated according to the following equation.

(5) $$f_{1}=\{[\sum_{t=1}^{n}\left|R_{t}-T_{t}\right|]/[\sum_{t=1}^{n}R_{t}]\}\times 100$$

## 5. Conclusions

A generic tablet formulation, similar to famotidine chewable tablets containing antacids, was developed and compared with pulverized reference chewable tablets to assess its pharmaceutical equivalence. Comparative dissolution tests were conducted, and the pulverized reference chewable tablets were used as the comparator product rather than an intact chewable tablet. According to previous literature regarding the particle size distribution of pulverized formulations, particles passing through a 12-mesh sieve were selected as the reference for comparative dissolution testing. The evaluation of the similarity factor showed that the test drug and the pulverized reference chewable tablet exhibited similar dissolution patterns, confirming that the two products were pharmaceutically equivalent.

The optimal particle size for pharmaceutical equivalence tests, including bioequivalence and comparative dissolution tests, is not yet well defined. As demonstrated, the intact chewable tablets and their pulverized forms exhibited different dissolution patterns, which may lead to differences in pharmacological outcomes. Therefore, regulatory agencies are encouraged to establish guidelines regarding the particle size distribution of pulverized tablets in comparative dissolution tests, especially when chewable tablets are used as reference products.

## Figures and Tables

**Table 1:** Active pharmaceutical ingredients of chewable tablets available in Korea.

API *	Market **	Pharmacological Efficacy	No. of Products
Alginic Acid 200/Carboxymethylcellulose Sodium 100	Export only	Adjuvant therapy in weight loss	1
Cyproheptadine Orotate Hydrate 0.75/DL-Carnitine Hydrochloride 75/L-Lysine Hydrochloride 75/Cyanocobalamin 0.5	OTC	Appetite stimulant	1
Dimenhydrinate 20/Scopolamine Hydrobromide Hydrate 0.125	OTC	Antihistamine	1
Dried Aluminium Hydroxide Gel 80/Magnesium Silicate 20/Scopolia Extract 5	Export only	Gastritis	1
Hydrotalcite 259/Glutamine 140/Soluble Azulene 2	OTC	Gastritis	1
Lamotrigine 2, 5, 25, 50, 100	ETC	Seizure	5
Meclizine Hydrochloride Hydrate 12.5/Scopolamine Hydrobromide Hydrate 0.125	OTC	Antihistamine	1
Microencapsulated Acetaminophen 86	OTC	Analgesics	1
Montelukast Sodium 4, 5	ETC	Antihistamine	115
Montelukast Sodium 5.2/Levocetirizine Hydrochloride 5.0	ETC	Antihistamine	1
Sildenafil 25, 50, 100	ETC	Erectile dysfunction	7
Simethicone 80	OTC	Gastrointestinal disturbance	2
Simethicone Powder 181.2/Loperamide Hydrochloride 2.0	OTC	Gastrointestinal disturbance, Diarrhea	1
Sodium Alginate 200, 250	ETC	Gastritis	2
Sucroferric Oxyhydroxide 2500	ETC	Serum phosphate regulation	1
Tadalafil 5, 10, 20	ETC	Erectile dysfunction	1

* Numbers are mg in formulation. ** OTC, over-the-counter; ETC, ethical drug. Products that contain multivitamins are excluded.

**Table 2:** Results of UV analysis in dissolution media.

Parameter		UV Absorption in Dissolution Medium			
		pH 1.2	pH 4.0	pH 6.8	Water
System suitability	Mean	0.0029	0.0079	0.0018	0.0017
	SD	0.0003	0.0002	0.0002	0.0004
	RSD (%)	0.10	0.07	0.08	0.18
Specificity	Blank	−0.0001	0.0001	0.0000	0.0001
	Placebo	0.0016	0.0005	0.0011	0.0013

**Table 3:** Results of calibration curves and validation parameters.

Parameters	Dissolution Medium			
	pH 1.2	pH 4.0	pH 6.8	Water
Slope	0.0310	0.0303	0.0252	0.0244
y-intercept	0.0010	−0.0001	0.0012	0.0057
SD of y-intercept	0.0005	0.0004	0.0012	0.0003
R^2^, coefficient of determination	1.0000	1.0000	0.9999	0.9978
Range (µg/mL)	0.509~12.212 (4.6~109.9% ^§^)	0.511~12.252 (4.6~110.3%)	0.503~12.064 (4.5~108.6%)	1.223~12.229 (11.0~110.1%)
Detection limit * (µg/mL)	0.045	0.047	0.157	0.042
Quantitation limit * (µg/mL)	0.145	0.144	0.478	0.130
Accuracy (Recovery) (%)	99.041 ± 2.070	98.386±1.413	99.432 ± 1.439	99.278 ± 2.310
Precision (%)				
repeatability	99.959 ± 0.471	99.515 ± 0.826	100.784 ± 0.853	99.929 ± 2.005
reproducibility	100.266 ± 0.513	99.825 ± 0.665	100.162 ± 0.879	100.766 ± 1.658

^§^ Calculated based on the released amount from the tablet. * Detection limit = 3.3σ/S. Quantitation limit = 10σ/S. Data were presented as mean ± standard deviation.

**Table 4:** Comparison of difference (*f*_1_) and similarity factor (*f*_2_).

Dissolution Medium	Pulverized Reference Tablet (PCT) vs. Test Tablet (FT)		Chewable Reference Tablet (CT) vs. Test Tablet (FT)		Chewable Reference Tablet (CT) vs. Pulverized Tablet (PCT)	
	*f*_1_	*f*_2_	*f*_1_	*f*_2_	*f*_1_	*f*_2_
pH 1.2	8.48	56.81	38.02	25.27	38.02	28.53
pH 4.0	10.03	53.10	27.76	36.05	27.76	31.02
pH 6.8	5.64	67.58	61.36	27.04	56.77	28.24
Water	4.28	75.95	48.25	34.19	45.18	36.24

**Table 5:** Various forms of chewable tablets as reference drugs.

Pharmaceutical Tests	API	Comparison	Reference
in vivo	pyrantel	either to swallow whole, to chew and swallow, or to swallow previously pulverized tablets	Wesche 1994 [6]
	raltegravir	administered after crushing	Krogstad 2021 [7]
	albendazole	crushed to a fine powder by mortar and pestle	Sawatdee 2019 [8]
	montelukast	intact chewable tablet with 240 mL water	Cánovas 2011 [9]
	montelukast	chewable tablet with warm water 240 mL	Zhu 2023 [10]
	montelukast	chewable tablet with 240 mL water	Zaid 2015 [11]
	sildenafil	chewable tablet with 50 mL water	Yoo 2017 [12]
	sildenafil	chewed until full disintegration and then swallowed with or without 250 mL water	Valenzuela 2011 [13]
	sildenafil	chewed and immediately swallowed	Marcelín-Jiménez 2012 [14]
	montelukast	intact chewable tablet	Knorr 2010 [15]
in vitro dissolution	verapamil	whole tablet, crushed tablet using a commercial tablet crusher, or tablet chewed in the mouth and then expelled into dissolution fluid	El-Gazayerly 2004 [4]
	lanthanum carbonate	whole tablet, crushed tablet, or the tablet fraction of less than 200 μm	Yang 2013 [3]
	montelukast	intact chewable tablet compared to ODT	Chen 2015 [16]
	albendazole	intact chewable tablet	Sawatdee 2019 [8]
	albendazole	intact chewable tablet	Kimaro 2019 [17]
	ciprofloxacin	intact chewable tablet	Usmani 2023 [2]

## Data Availability

Data is contained within the article.
